# Cognitive reserve predicts episodic memory enhancement induced by transcranial direct current stimulation in healthy older adults

**DOI:** 10.1038/s41598-024-53507-0

**Published:** 2024-02-28

**Authors:** Marco Sandrini, Rosa Manenti, Elena Gobbi, Ilaria Pagnoni, Andrea Geviti, Cristina Alaimo, Elena Campana, Giuliano Binetti, Maria Cotelli

**Affiliations:** 1 School of Psychology, University of Roehampton, London, UK; 2 Neuropsychology Unit, IRCCS Istituto Centro San Giovanni di Dio Fatebenefratelli, Brescia, Italy; 3 Statistics Service, IRCCS Istituto Centro San Giovanni di Dio Fatebenefratelli, Brescia, Italy; 4 MAC Memory Clinic and Molecular Markers Laboratory, IRCCS Istituto Centro San Giovanni di Dio Fatebenefratelli, Brescia, Italy

**Keywords:** Neuroscience, Psychology

## Abstract

Episodic memory shows the largest degree of age-related decline. Anodal transcranial Direct Current Stimulation (tDCS) can enhance episodic memory in aging but there is also evidence of response variability even when using identical stimulation parameters. To explore which inter-individual factors (i.e. age, education, encoding performance, cognitive reserve, tDCS group and timing of tDCS application) may directly and/or indirectly modulate verbal memory recall, we used data from our previous tDCS studies that showed enhanced episodic memory recall in 80 healthy older adults. In these studies we used the same paradigm and stimulation parameters but tDCS was applied during different memory stages. Memory recall was tested 48 hours and 30 days after encoding. Univariate regression models showed that tDCS group (Anodal vs. Sham) predicted memory recall, indicating higher scores in the Anodal group than in the Sham group. Encoding performance predicted memory recall in both tDCS groups. Multiple regression models revealed that cognitive reserve, measured with a life experience questionnaire, predicted memory recall only for the Anodal group. Higher cognitive reserve was linked to better memory recall. Accounting for individual differences in cognitive reserve at baseline helps to explain tDCS responsiveness. This knowledge may contribute to optimize its use in older adults.

## Introduction

Episodic memory is a type of long-term memory that involves the recollection of past events or experiences^1^. This memory declines with age and is typically the first symptom reported by patients suffering from Alzheimer's disease (AD). Given the worldwide increase in the proportion of older adults, the development of interventions against age-related episodic memory decline is of great scientific and public interest^2^.

Anodal transcranial Direct Current Stimulation (tDCS) is a safe, non-invasive brain stimulation technique^3^ that may enhance episodic memory in healthy older adults^4^ and individuals with amnestic mild cognitive impairment (aMCI) and mild AD^5,6^. aMCI is considered the prodromal stage of AD^7^.

Both local and global neural effects have been previously proposed as plausible mechanisms of this memory enhancement. Anodal tDCS increases neuronal excitability by causing a depolarization of the resting membrane potential^3,8^. Thus, Anodal tDCS may induce neuroplasticity through changes in synaptic plasticity^8^. Anodal tDCS modifies brain neurometabolism as well^9^. There is evidence that age modulates the effect of Anodal tDCS on metabolite concentration. Antonenko et al.^10^ showed a reduction of GABA levels after Anodal tDCS relative to Sham stimulation, reflecting the preserved neuromodulatory effect of tDCS in older adults. In addition to these local effects, Anodal (Active) tDCS induces functional changes by dynamic modulation of functional connectivity^11^.

Our studies in healthy older adults have shown enhanced delayed verbal episodic memory recall with Anodal tDCS applied over the left lateral prefrontal cortex (PFC), a critical node in the episodic memory network^12^, during encoding^13^, immediately after encoding (i.e., consolidation, the processes that stabilize memories after encoding, transforming them into long-term memory)^14^ or after a contextual reminder (i.e., conceivably through reconsolidation, the processes that re-stabilize the consolidated memories after reactivation)^15^. There are concerns regarding the high variability in the memory stimulation effects^5,16^. While the variability may be explained partly by stimulation parameters (timing of application, intensity, duration, number of sessions, location, electrodes montage, dimension of electrodes), inter-individual differences may also contribute to the heterogeneity of tDCS outcome^17,18^. To date, it remains unclear which individual factors are able to predict the effects of Anodal tDCS on episodic memory enhancement in the elderly population and thus help to explain inter-subject variability of tDCS responsiveness^19^. Some evidence has been published about the influence of baseline cognitive function^20,21^ and education^22,23^ on the effects of tDCS on memory in healthy older adults. As education is a proxy indicator of cognitive reserve (CR)^24^, this result suggests that tDCS responses are better for older adults with higher CR. However, using a single proxy indicator may not reflect the CR concept appropriately, since CR is a complex construct and it is determined by various components. The three main sources of CR correspond to the three main aspects of an individual’s life experience: (a) education level^25^, (b) work-related activities^26^, and (c) leisure time^27^. CR could be a protective factor in subjects at risk of cognitive decline, optimizing cognitive performance despite brain changes^24,28^. Epidemiologic studies strongly support the notion that higher levels of CR are associated with better cognitive performance, as well as a reduced risk of developing dementia later in life^29^. The hypothesis underlying the concept of CR is that individual differences in the way tasks are processed provide a reserve against brain pathology^30^.

In a recent study, we reanalysed the data acquired in our previous studies with Subjective Memory Complaints (SMC) and aMCI participants with the aim of investigating how the tDCS-induced reconsolidation effects could be modulated by individual factors such as age, CR, education level, diagnosis and encoding performance in these AD-risk populations. Our main finding was that the higher leisure time subscore of the Cognitive Reserve Index questionnaire (CRI-q^31^) predicted better delayed retrieval performances, but none of the individual factors analysed modulated the tDCS-induced memory enhancement effects, indicating that the effects of the predictors on retrieval performance occurred regardless of the tDCS group (Anodal vs. Sham)^32^.

Aiming to explore whether the effects of Anodal tDCS on episodic memory recall in healthy older adults could be directly and/or indirectly modulated by individual factors such as age, CR, education level, encoding performance, tDCS group and the timing of tDCS application (i.e., during encoding, consolidation or reconsolidation), we used the data from our tDCS studies that used the same paradigm and stimulation parameters but with tDCS applied during different memory stages^13–15^.

## Methods

**Figure 1:**
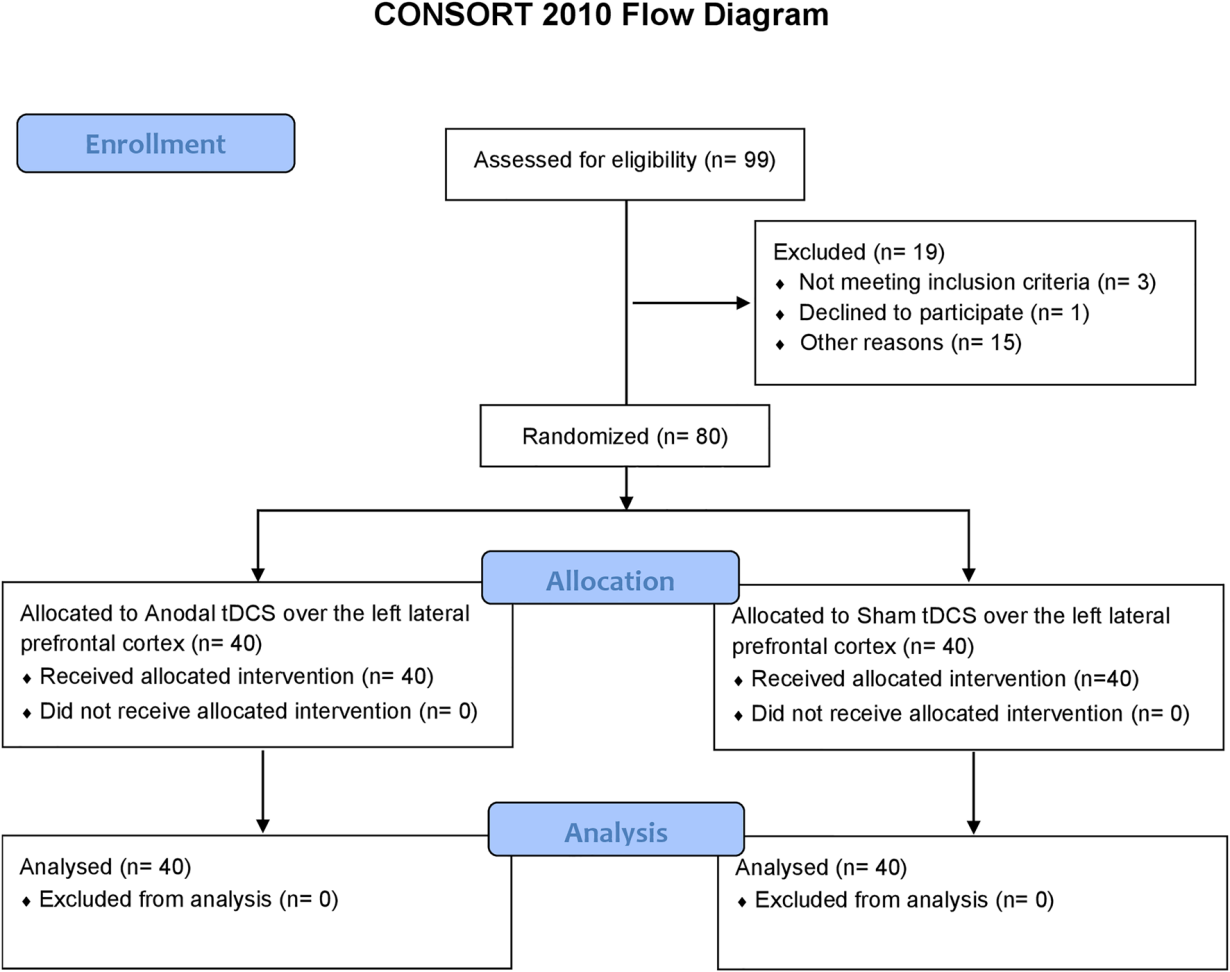
Consort flow diagram. The flow diagram displays the progress of all participants through the study.

Recruitment and tDCS protocol have been conducted at the IRCCS Istituto Centro San Giovanni di Dio Fatebenefratelli of Brescia from October 15th, 2013 to November 19th, 2018 (see Fig. 1).

### Participants

Data from 80 healthy older adults (females 57, males 23; mean age = 67.9, SD = 5.0 years; mean education = 12.0, SD = 4.4 years) were considered in this analysis (Table 1).

**Table 1:** Demographical, clinical and neuropsychological data.

	Anodal tDCS (n = 40)	Sham tDCS (n = 40)	Cut-off
Age (years)	68.3 (5.3)	67.6 (4.7)	
Gender (male/female)	13/27	10/30	
Education (years)	12.2 (4.3)	11.8 (4.4)	
EHI (%)	84.9 (17.1)	87.6 (14.3)	
Geriatric depression scale (GDS)	4.2 (4.5)	5.4 (4.9)	< 11
STAI-state anxiety inventory	39.3 (8.5)	37.6 (10)	
STAI-trait anxiety inventory	38.4 (8.5)	40.1 (6.6)	
Everyday memory questionnaire (EMQ)	42.2 (10.9)	39.9 (11.3)	
**Cognitive reserve index—questionnaire (CRI—q)**			
CRI—total score	119.3 (17.6)	120.3 (18.1)	
CRI—education	111.1 (13.8)	111.8 (14.5)	
CRI—working activity	106 (15.9)	106.2 (17.8)	
CRI—leisure time	124.9 (23.7)	128 (19.5)	
**Screening test for global cognition**			
MMSE	29.1 (1)	29 (0.9)	≥ 24
**Non-verbal reasoning**			
Raven’s coloured progressive matrices	30.4 (3.8)	29.8 (4.4)	> 17.5
**Memory**			
Digit span (forward)	6.1 (1.2)	5.9 (0.9)	> 4.25
Story recall	13.9 (3.4)	13.9 (3.6)	> 7.5
RAVLT, immediate recall	49.4 (8.1)	46.7 (7.4)	> 28.52
RAVLT, delayed recall	10.6 (2.8)	9.9 (2.5)	> 4.68
Rey-Osterrieth complex figure, recall	16.4 (5.7)	15.4 (5.3)	> 9.46
**Language**			
Verbal fluency, phonemic	41.5 (11.5)	40.1 (11.3)	> 16
Verbal fluency, semantic	48.6 (8.7)	46.4 (8.6)	> 24
**Praxis**			
Rey-Osterrieth complex figure, copy	32.3 (2.4)	31.7 (3.1)	> 28.87
**Attentional and executive functions**			
Trial Making Test, part A (seconds)	41.2 (17)	40.1 (14.3)	< 94
Trial Making Test, part B (seconds)	113.6 (46.2)	113.8 (46.1)	< 283
Trial Making Test, B-A (seconds)	72.4 (39.4)	71.9 (39.7)	< 187

Raw scores are reported (SD in brackets). Cut-off scores according to Italian normative data are reported.

*EHI* Edinburgh handedness inventory, *MMSE* Mini Mental State Examination, *RAVLT* Rey Auditory Verbal Learning Test, *tDCS* transcranial Direct Current Stimulation.

All the subjects included in the previous studies underwent a standardized protocol, which included an initial clinical and neuropsychological assessment and an experimental memory task with Anodal or Sham tDCS. The participants were native Italian speakers and had normal or corrected-to-normal vision. Participants were excluded from the studies if they had a history of alcohol abuse or major neurological or psychiatric disorders. Moreover, individuals in which any contraindication to tDCS was noted were not included. Before being recruited, healthy older adults completed a detailed clinical and neuropsychological assessment, carried out by an expert neuropsychologist, in order to ensure the absence of any cognitive deficit. The presence of pathological score in one or more neuropsychological tests was an exclusion criterion. The neuropsychological battery included tests for assessment of global cognition (Mini Mental State Examination, MMSE ^33^), nonverbal reasoning (Raven's Colored Progressive Matrices^34^), verbal fluency (phonemic and semantic^35^), visuospatial ability (Rey-Osterrieth's Complex Figure-ROCF, copy^36^), attention and executive functions (Trail Making Test-TMT, part A and part B^37^). In addition, all participants underwent to an extensive memory assessment (story recall^38^, ROCF, recall^36^, Digit Span forward test^39^, Rey Auditory Verbal Learning Test-RAVLT, immediate and delayed recall^40^). With regard to clinical assessment, subjective memory complaints were assessed using the 20-item version of the Everyday Memory Questionnaire (EMQ)^41,42^. Furthermore, the trait and state anxiety were measured with the State-Trait Anxiety Inventory-STAI, a 40-item self-report questionnaire^43,44^ and depressive symptoms were assessed using the 30-item version of the Geriatric Depression Scale (GDS)^45,46^. Moreover, the Cognitive Reserve Index questionnaire (CRI-q) was administered to all the healthy older adults. The CRI-q evaluates the cognitive reserve of an individual by means of the compilation of information relating to a person’s lifetime and was constructed based on the main cognitive reserve indices proposed by Stern^28^. In addition to the collection of biographical information, the questionnaire consists of three subscales that investigate three different domains of life: CRI-Education, CRI-Working Activity and CRI-Leisure Time. The CRI-Education subscale investigates the years of education and the possible training courses; the CRI-Working Activity subscale assesses the level of adulthood professions and the CRI-Leisure Time subscale evaluates the various intellectual activities, social activities, physical activities and the number of children during the person’s lifetime. An index is calculated for each of these subscales and the average of these subscores consists in a final total score (CRI-Total Score), which can be classified over five levels: low (less than 70), medium–low (70–84), medium (85–114), medium–high (115–130) and high (more than 130)^31^ (see Table 1).

Regardless of the timing of tDCS application (during encoding (ENC), during consolidation (CON) or during reconsolidation (REC)) the previously recruited participants were randomized in a 1:1 ratio into two groups: Anodal tDCS (anode over the left lateral prefrontal cortex and cathode over right supraorbital area) or Sham tDCS. Each participant was randomly assigned to the two groups according to MMSE score^33^ and age. In Sandrini et al.^15^, we included a third group receiving Anodal tDCS without reactivation of memories that was not included in the present manuscript.

Stratified randomization is achieved by generating a separate block for each combination of covariates and participants were assigned to the appropriate block of covariates by a researcher blinded to the study aims. Details of the allocated group were given on cards contained in sequentially numbered, opaque and sealed envelopes. The study protocol was executed with no significant changes from the beginning.

The experimental methodologies obtained ethical approval from the local Human Ethics Committee of IRCCS Istituto Centro San Giovanni di Dio Fatebenefratelli, Brescia, and the protocol was conducted in accordance with the Declaration of Helsinki and recorded according to CONSORT guidelines (see Table S1, CONSORT 2010 checklist)^47,48^. The trial was not registered. Each participant was informed about the procedures and only after a safety screening on the possible risks of tDCS, a written informed consent was obtained.

### Memory task procedure

Experimental data collection across studies was supervised by the same investigator (RM) in order to ensure compliance with the standardized protocol. In each of the previous studies, the experimental procedure included learning and retrieval sessions on different days^13–15^. In all studies, on Day 1 (encoding/learning session), participants were asked to memorize a list of 20 concrete words, selected from the *“Corpus e Lessico di Frequenza dell’Italiano Scritto (CoLFIS)”*^49^, balanced by length, frequency, familiarity and image ability. The words were written on pieces of cardboard and taken one at a time from a white bag. Participants were asked to read and pay attention to each word and then place the cardboards in a blue bag. After this procedure was completed, the experimenter asked participants to recall orally as many words as possible. Before the next round, all the cardboards were placed and mixed in the white bag. The learning session consisted in a maximum of five rounds or until participants could recall at least 85% of the words (17 out of 20 words). Moreover, at the end of the encoding/learning session, all participants completed a semi-structured memory strategies questionnaire to assess the possible strategies used during the learning session in order to investigate their influence on subsequent memory recall. Participants had to assign a score from 1 to 10 (1 = never, 10 = always) to each strategy according to how often they had used each strategy. The 12 listed strategies were: (1) to use words’ initials, (2) to create sentences including some of the presented words, (3) to imagine the pictures corresponding to the presented words, (4) to repeat the words, (5) to create songs including some of the presented words, (6) to create rhymes between the displayed words, (7) to translate the words in a foreign language, (8) to create associations of words, (9) to create a brief story including the presented words, (10) to associate each word to a personal event, (11) to classify each word as easy or difficult, abstract or concrete, positive or negative, and so forth, (12) to imagine the words’ sound, color, shape, and so forth, and (13) other strategies^13–15,50^. The encoding session lasted approximately 25–30 min.

For the retrieval sessions the procedure is the same among studies. At the end of Day 1, each participant was not given details of the next sessions and for this reason, subjects could not expect a memory test. So, after 48 h and 30 days (Day 3 and Day 30 respectively from Day 1-encoding session), in the same room of Day 1, the experimenter asked the participants to recall orally as many words as possible from Day 1, and the experimenter noted the remembered words (primary outcomes). When participants reported that they could not remember any more words, the experimenter asked them to perform a figure-copying task for 30 s. This procedure was repeated for four consecutive recall rounds in order to test the reliability of the recall. The recall session lasted 15 minutes.

**Figure 2:**
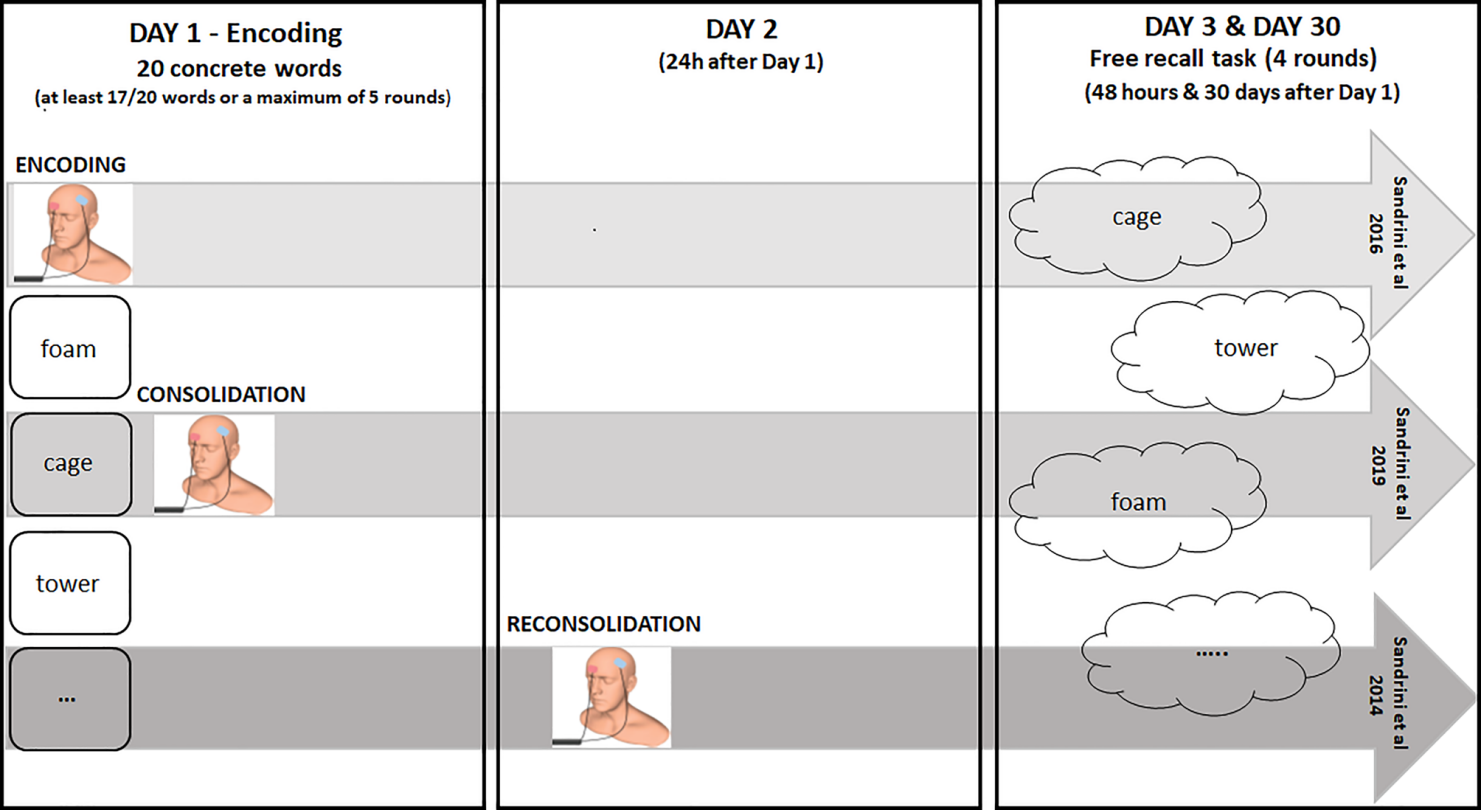
Experimental paradigm. In all studies, at the Day 1 (encoding session), participants were asked to memorize a list of 20 concrete words (at least 17/20 words or a maximum of 5 rounds). The experimenter asked participants to remember orally as many words as possible after each round. For the retrieval sessions the procedure is the same among studies. After 48 h and 30 days (Day 3 and Day 30) from the encoding session, the experimenter asked the participants to recall the words memorized during the Day 1 for 4 rounds (free recall task). The only difference between the studies concerned the timing of the tDCS application. Sandrini et al.^13^ applied tDCS during the encoding session (Day 1), whereas Sandrini et al.^14^ applied tDCS immediately after the encoding session (i.e., during consolidation). Otherwise, in the study of Sandrini et al.^15^, 24 h after the encoding tDCS was applied after reactivation of existing memories (i.e., during reconsolidation) (Day 2).

The only difference between the studies included in this analysis concerned the timing of the tDCS application. In the study of Sandrini et al.^15^, in addition to the sessions described above, 24 h after encoding tDCS was applied after reactivation of existing memories (i.e., during reconsolidation, REC) (Day 2). In other words, in the same room of the Day 1, the experimenter presented to participants the empty blue bag and asked: *“Do you remember this blue bag and what we did with it yesterday?”.* Participants had to describe the procedure without reporting the words learned. Existing episodic memories are automatically reactivated if the original spatial context (i.e., the same experimental room of Day 1) is part of the reminder^51^. Ten minutes after the reactivation, subjects received tDCS. It has been shown that reconsolidation begins between 3 and 10 min after memory reactivation^52^. Otherwise, Sandrini et al.^13^ applied tDCS during the encoding (ENC) session (Day 1), whereas Sandrini et al.^14^ applied tDCS immediately after the encoding session (i.e., during consolidation, CON) (Day 1) (Fig. 2).

### Transcranial Direct Current Stimulation (tDCS) procedure

The tDCS procedure was the same in all studies considered and participants could receive Anodal or Sham tDCS^13–15^. The tDCS was applied in accordance with safety guidelines^53,54^.

Stimulation was applied with a battery-powered constant current stimulator (BrainStim, EMS; Bologna, Italy) and a pair of rubber electrodes covered with sponges (7 cm × 5 cm) soaked in saline solution. The same stimulator was used in all studies and the impedance of the electrodes was verified before stimulation started. The impedance level was kept below 5 kΩ; if it increased during stimulation, the stimulator automatically stopped the current delivery. In order to optimize the impedance levels, each side of the sponges was soaked in saline solution through a disposable plastic syringe^55,56^. Since we applied two 35 cm^2^ sponges, about 12 ml of solution was used^56^. The electrode placement was conducted according to the 10–20 electroencephalogram international system^57^ in order to ensure the consistent placement of electrodes on heads of different sizes and shapes, measuring the distance between some landmarks (i.e. inion and nasion, and left and right preauricular points)^55^. In all studies, the anode was placed over F3 and the cathode over the contralateral supraorbital region^13–15, 21,58,59^. There is evidence that for F3 the main targeted region was Brodmann area 9 within the left lateral PFC^60^. Once these locations were identified, the electrodes were affixed to the head using elastic straps in order to avoid the displacement of the electrodes over the course of a tDCS session and any change of the current distribution during the stimulation^61^. In all studies we used elastic straps of the same size. Stimulation was administered in Anodal or Sham mode using blind number codes previously entered into the device, so neither the examiner nor the subjects knew which tDCS stimulation was applied. During Sham mode, the display imitated the settings of the Anodal mode by simulating typical parameters of current strength, voltage, and impedance. The operator could not notice any difference between Anodal and Sham stimulation. The Anodal tDCS condition involved the application of current for 15 min at an intensity of 1.5 mA with a 10-s ramp at the beginning and at the end of the tDCS session. For the Sham condition, the current was turned off 10 s after the start of stimulation and turned on again for 10 s at the end of the stimulation period^13–15,21,32,58,59^. The current density (0.043 mA/cm^2^) was kept below the safety limits^53,54^. At the end of the tDCS session, all participants were asked to complete a questionnaire to assess perceptual sensations and side effects induced by tDCS^62^ (i.e. itching, pain, burning, heat, pinching, iron taste, fatigue, effect on performance, through a 5-point-scale: 0 = none, 1 = mild, 2 = moderate, 3 = considerable, and 4 = strong).

### Statistical analysis

The Gaussian distribution of the dependent variables was checked through graphical examination and the Shapiro–Wilk test. The variable “Free Recall Day 3” was normally distributed, while “Free Recall Day 30” displayed a right-skewed distribution and was analysed with generalized linear models. A set of predictors was chosen (based on former study evidence^17,32^), thus inference on them did not need to be adjusted for multiple testing^63,64^.

The direct effect of each predictor on the response variables was assessed using univariate regression models. The direct effect of age, education, total and subscales CRI scores, timing of tDCS application (Encoding vs. Consolidation vs. Reconsolidation), tDCS group (Anodal vs. Sham) and encoding performance (i.e., number of words recalled during the last round of the encoding session) on the response variables was evaluated.

A linear model was used for “Free Day Recall 3”, while a generalized linear model with Tweedie distribution and log-link function was applied to “Free Day Recall 30”. This type of distribution is particularly suited for right-skewed data including a mass of zero values.

The potential interaction effects between the tDCS group and the other predictors on the response variables were investigated through multiple regression models. For each model, a single predictor, tDCS group, and the interaction of this predictor with the tDCS group were inserted as independent variables. All models were adjusted for age.

The goodness of fit of each model was evaluated through the Akaike Information Criterion (AIC; lower values denote a better fit). Perceptual sensations induced by tDCS and strategies used during encoding were compared between the Anodal and the Sham groups using Mann–Whitney U-test. Statistical analyses were performed using SPSS version 28 (Dell Software, Aliso Viejo, CA, United States). Statistical significance was set at p < 0.05.

## Results

The strategies more frequently reported by the participants after encoding were: to imagine the pictures corresponding to the words displayed; to repeat the words; to create associations of words; and to associate each word to a personal event. Moreover, none of the strategies showed significant differences between Anodal and Sham groups (p > 0.05).

By interpreting the questionnaire completed by all subjects at the end of each type of stimulation, we inferred that all the subjects tolerated well the stimulation and no side effects were reported^62^. Only marginal perceptual sensations were reported in Anodal and Sham groups: itching and pinching were the most commonly reported perceptual sensations, with light to moderate intensity. Overall, the experienced perceptual sensations started at the beginning of the experiment and quickly disappeared.

Regarding the data acquired using the questionnaire to assess the perceptual sensations induced by tDCS, the scores reported by the Anodal and Sham groups were not different (ENC: Anodal tDCS group: 1.2, SD 0.6, Sham tDCS group: 1, SD 0.6; p = 0.43; REC: Anodal tDCS group: 1.8, SD 1.5, Sham tDCS group: 1.3, SD 0.8; p = 0.33; CON: Anodal tDCS group: 2.1, SD 1.4, Sham tDCS group: 1.9, SD 0.8; p = 0.63). Hence, there are no reasons to reject the blinded character of this study on the basis of these results.

Results of univariate models are presented in Tables 2 and 3. A significant effect of the tDCS group (Anodal vs. Sham) was found on both the response variables “Free Recall Day 3” and “Free Recall Day 30” (Day 3: β = 2.99, p < 0.001, AIC = 440.5; Day 30: β = 0.58, p < 0.001, AIC = 440.8), indicating higher performance scores in the Anodal group than in the Sham group on Day 3 and on Day 30 (positive β). See Supplementary Table S2 for free recall data.

**Table 2:** Free recall day 3.

Independent variables/predictors	Beta coefficient	*p*-value	AIC
tDCS group	**2.99**	** < 0.001**	**440.5**
Education	**0.221**	**0.025**	**448**
CRI-total score	0.038	0.121	450.5
CRI-education	0.041	0.180	451.1
CRI-working activity	0.024	0.361	452
CRI-leisure time	0.026	0.201	451.2
tDCS timing		0.630 (Global)	453.9
	− 1.046 (REC vs. CON)	1 (REC vs. CON)	
	− 0.563 (ENC vs. CON)	1 (ENC vs. CON)	
	0.484 (ENC vs. REC)	1 (ENC vs. REC)	
Encoding performance	**0.631**	** < 0.001**	**435.7**
Age	− 0.076	0.387	452.1

*tDCS Group* sham reference category, *CRI* cognitive reserve index, *ENC* during encoding, *CON* during consolidation, *REC* during reconsolidation.

Significant results shown in bold. P-values of pairwise comparisons for tDCS Timing were adjusted using Bonferroni correction.

**Table 3:** Free recall day 30.

Independent variables/predictors	Beta coefficient	*p*-value	AIC
tDCS group	**0.578**	** < 0.001**	**440.8**
Education	0.021	0.186	457.5
CRI-total score	0.004	0.337	458.4
CRI-education	0.004	0.365	458.5
CRI-working activity	0.001	0.895	459.3
CRI-leisure time	0.003	0.413	458.6
tDCS timing		0.295 (Global)	458.9
	0.214 (REC vs. CON)	0.618 (REC vs. CON)	
	− 0.025 (ENC vs. CON)	1 (ENC vs. CON)	
	− 0.238 (ENC vs. REC)	0.484 (ENC vs. REC)	
Encoding performance	**0.114**	** < 0.001**	**439.1**
Age	0.005	0.737	459.2

*tDCS Group* sham reference category, *CRI* cognitive reserve index, *ENC* during encoding, *CON* during consolidation, *REC* during reconsolidation.

Significant results shown in bold. P-values of pairwise comparisons for tDCS Timing were adjusted using Bonferroni correction.

The number of words recalled during the last round of the encoding session predicted free recall performances both on Day 3 and on Day 30 (Day 3: β = 0.63, p < 0.001, AIC = 435.7; Day 30: β = 0.11, p < 0.001, AIC = 439.1), suggesting that higher encoding performance scores resulted in better free recall performance scores.

Education was a significant predictor only for Day 3 (β = 0.22, p = 0.025, AIC = 448), suggesting higher performance in delayed recall in subjects with a higher level of education.

For both response variables, the best predictor (displaying the lowest AIC) was the number of words recalled during the last round of the encoding session, i.e., encoding performance (Day 3: AIC = 435.7; Day 30: AIC = 439.1). In order to check for potential confounders in evaluating the relationship between encoding performance and Free Recall, a model was also built adding age, education and CR as controls. Only age was significantly associated with Free Recall, and only at Day 30 (Partial R^2^: Encoding Performance = 0.22; Age = 0.07).

**Figure 3:**
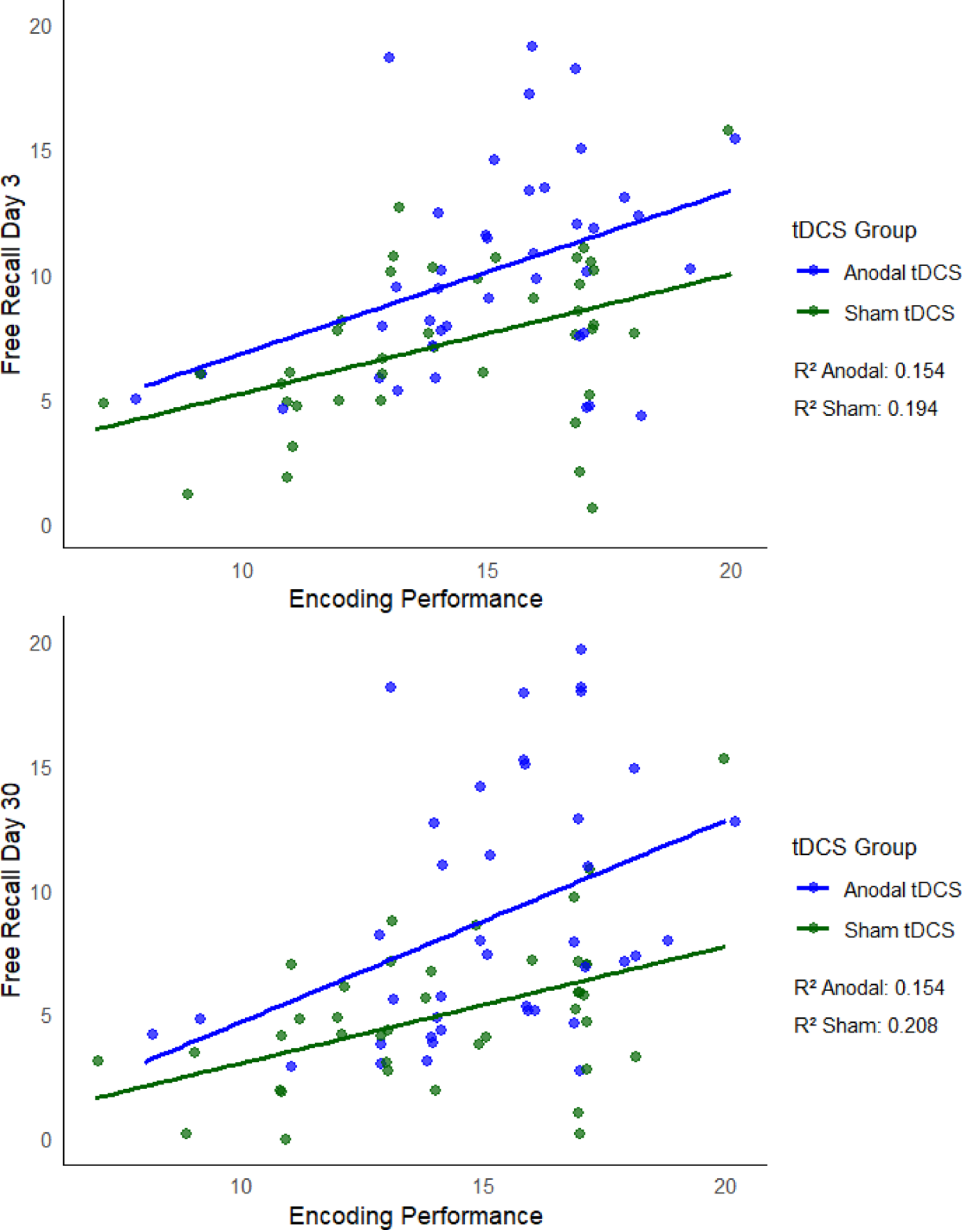
Association between response variables and encoding performance. The performance at the encoding session (number of words recalled during the last round of the encoding session) predicted free recall performances both on Day 3 and on Day 30, suggesting that higher encoding performance scores resulted in better free recall performance scores.

The association between response variables and encoding performance is graphed in Fig. 3.

**Figure 4:**
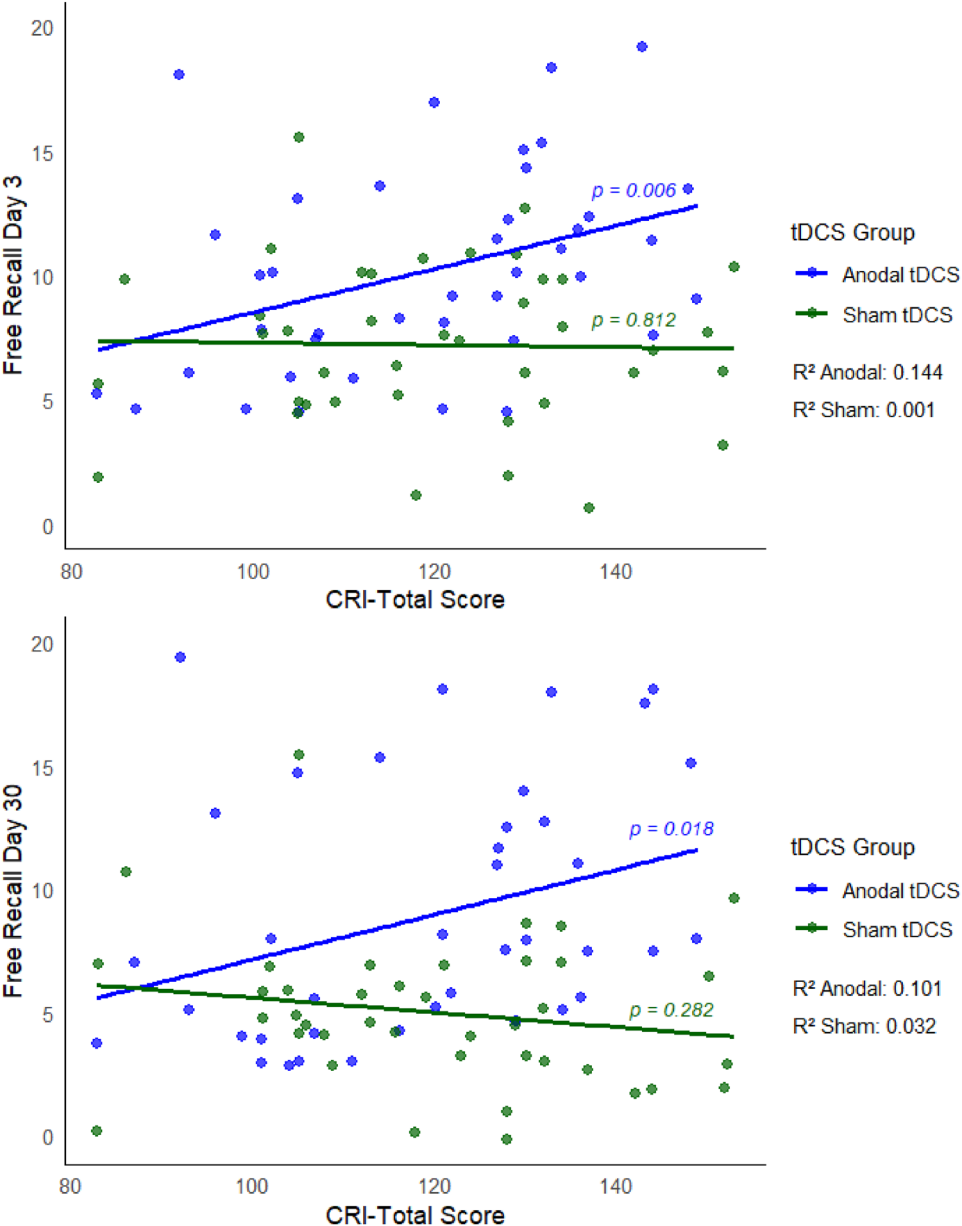
Interaction between group and CRI-Total Score. Multiple models revealed significant interaction effects of tDCS group with the CRI-Total Score. CRI-Total Score significantly predict free recall performance at the Day 3 and Day 30 only for subjects assigned to the Anodal tDCS group.

**Figure 5:**
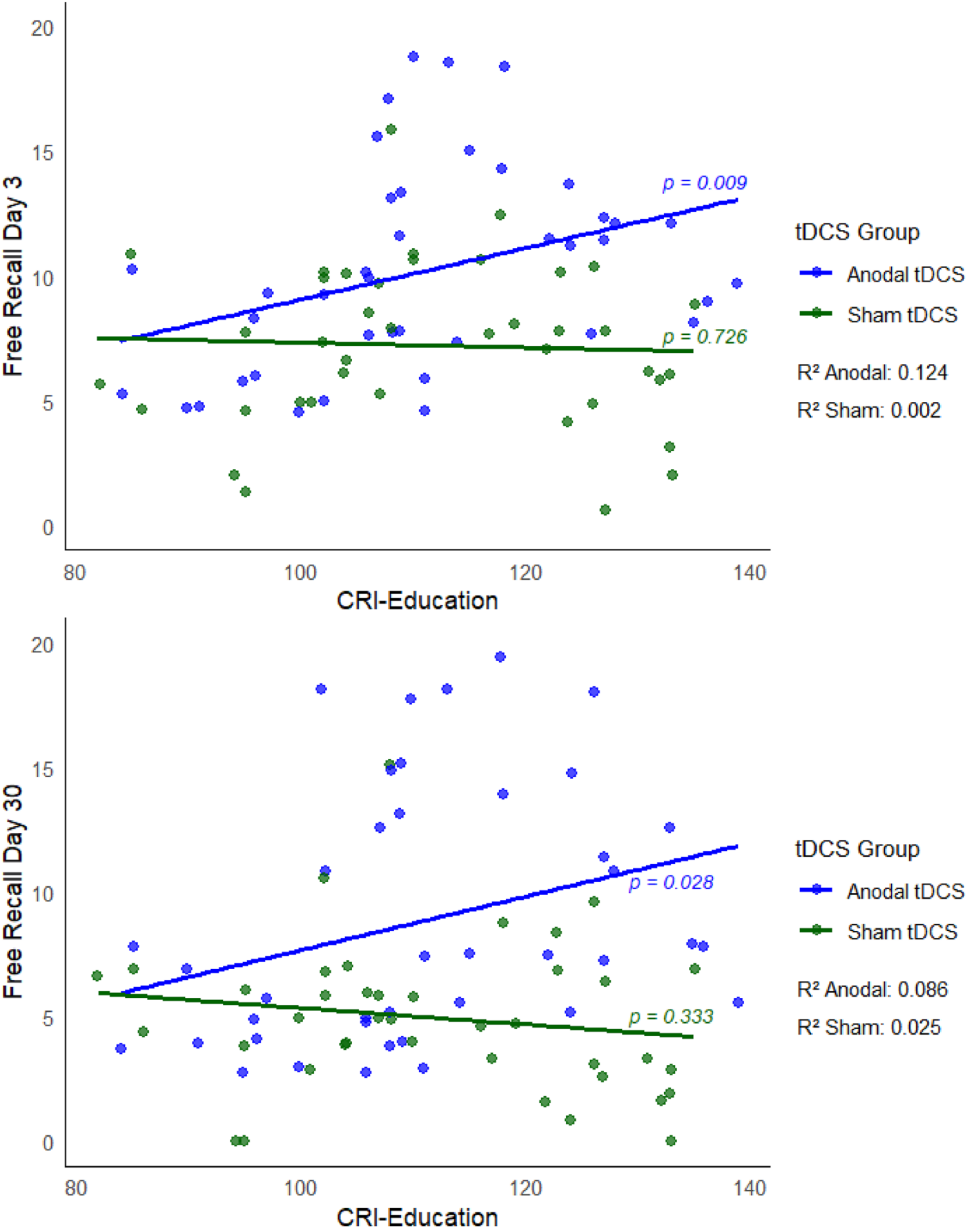
Interaction between group and CRI-Education. Multiple models revealed significant interaction effects of tDCS group with the CRI-Education subscale. CRI-Education significantly predict free recall performance at the Day 3 and Day 30 only for subjects assigned to the Anodal tDCS group.

Multiple regression models (Tables 4 and 5) revealed that, for both response variables, the interaction effects of the tDCS group with both the CRI-Total Score and the CRI subscale measuring education are significant [Day 3: p(CRI-Total Score) = 0.033, p(CRI-Education) = 0.034; Day 30: p(CRI-Total Score) = 0.020, p(CRI-Education) = 0.033]. These interactions (cross-over interactions) are presented in detail in Figs. 4 and 5 CRI-Total Score and CRI-Education significantly predict free recall performance only for subjects assigned to the Anodal tDCS group.

**Table 4:**
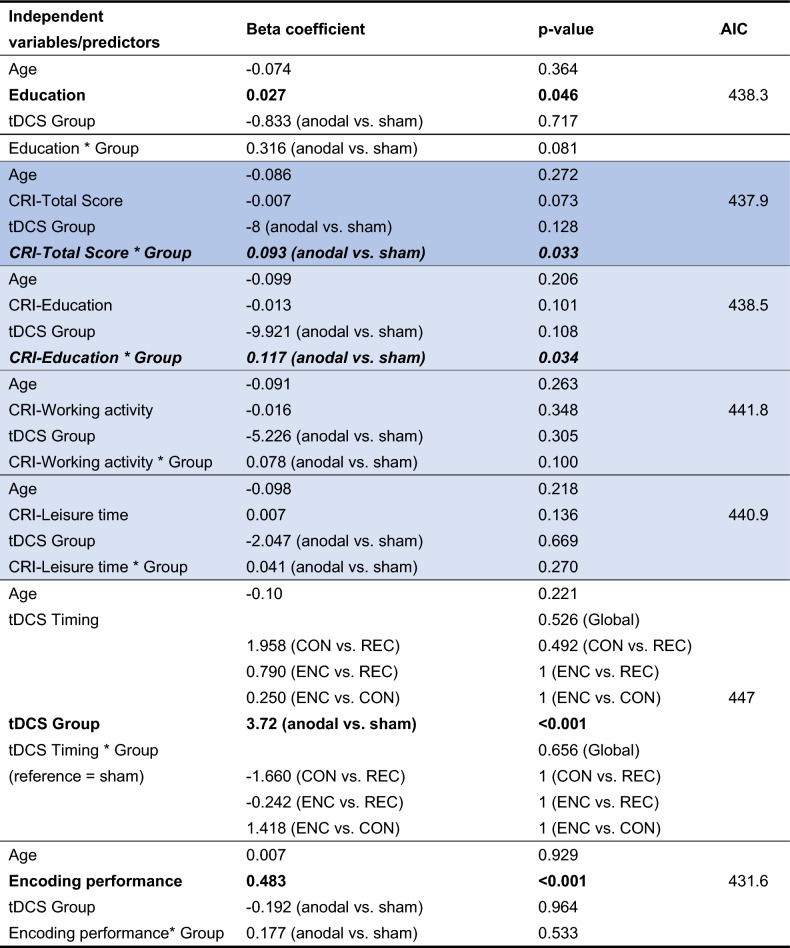
Free recall—Day 3.

The colored lines indicate models for CRI-Total Score (blue) and corresponding CRI subscale (light blue).

*CRI* cognitive reserve index, *AIC* Akaike information criterion, *ENC* during encoding, *CON* during consolidation, *REC* during reconsolidation.

Significant results are shown in bold. Significant interactions in italics. P-values of pairwise comparisons for tDCS Timing were adjusted using Bonferroni correction.

**Table 5:**
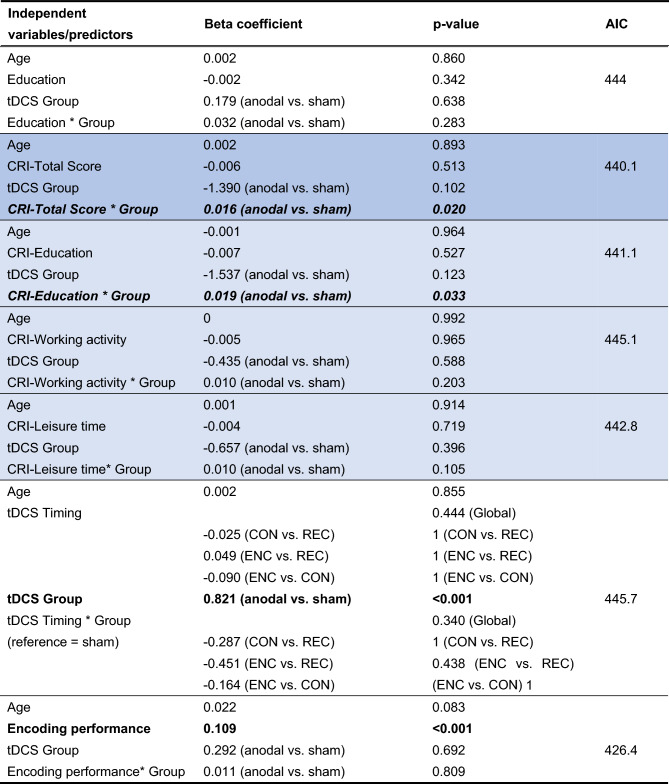
Free recall—Day 30.

The colored lines indicate models for CRI-Total Score (blue) and corresponding CRI subscale (light blue).

*CRI* cognitive reserve index, *AIC* Akaike information criterion, *ENC* during encoding, *CON* during consolidation, *REC* during reconsolidation.

Significant results are shown in bold. Significant interactions in italics. P-values of pairwise comparisons for tDCS Timing were adjusted using Bonferroni correction.

## Discussion

The aim of this work was to use data from our previous tDCS studies^13–15^ in order to explore the inter-individual factors that could influence the effect of Anodal tDCS on verbal episodic memory recall.

In particular, we investigated whether factors such as age, CR, education, timing of tDCS application (during encoding, consolidation or reconsolidation phase), tDCS group and encoding performance assessed on Day 1 could modulate the free recall performance tested on Day 3 and Day 30, respectively 48 h and 30 days after the encoding session.

The tDCS group (Anodal vs. Sham) significantly predicted the free recall performance both on Day 3 and Day 30, indicating higher performance scores in the Anodal tDCS group than in the Sham group. In detail, beta coefficients of Anodal vs. Sham were positive (2.99 and 0.58) and significantly different from zero (p < 0.001) for both recall on Day 3 and 30 (Tables 2 and 3). This result confirmed the possibility to enhance delayed verbal episodic memory recall with a single session of Anodal tDCS over the left lateral PFC in healthy older adults. The memory enhancement effect observed in our previous study with tDCS during encoding is in agreement with the hypothesis that a consolidation mechanism is susceptible to Anodal tDCS and contributes more to offline effects than online effects^65,66^. For the other previous studies with tDCS during consolidation or reconsolidation, facilitation of the consolidation processes might be the mechanism acting during the hours or days after tDCS^67^. The reactivation of encoded memories (or “replay”) in subsequent waking state^68^ may be critical for memory consolidation. tDCS applied during awake periods, such as during consolidation or reconsolidation, might boost neural reactivation and therefore enhance systems-level consolidation^67^. In addition, there is evidence that higher resting-state functional connectivity within the frontoparietal control network, specifically the left frontal cortex (LFC) hub, contributes to higher reserve^69^ and increased LFC connectivity is associated with higher reserve in the memory domain in normal and pathological aging^70^. Thus, tDCS over left PFC might have increased the LFC connectivity. Multiple regression models revealed that age, education level, tDCS timing and encoding performance did not modulate the tDCS-induced episodic memory enhancement. However, these analyses showed a significant interaction between the tDCS group (Anodal vs. Sham) and CRI-Total Score and between the tDCS group (Anodal vs. Sham) and the CRI-Education subscale. In the Anodal tDCS group, CRI-Total Score and CRI-Education score predicted free recall performance (both on Day 3 and Day 30): the higher the CRI scores, the better the free recalls. No effects were found in the Sham tDCS group.

Previous tDCS evidences found more working memory improvement in older adults with more years of education^22,23^. There is evidence that older adults with less education show greater declines in resting-state brain system segregation, as indexed by a measure of large-scale network organization and function^71^. However, the results of the current study showed that higher CRI-Education, not years of education, is associated with better memory recall in the Anodal group. CRI-Education includes not only the years of education but also considers each year of school failure and courses with educational characteristics (e.g., learning to play a musical instrument or learning a foreign language) carried out during adulthood for at least 6 months. A possible explanation might be the better sensitivity of CRI-Education compared to considering only years of education.

CRI-Total score provides a global proxy of CR based on a range of cognitively stimulating life experiences occurring throughout the lifespan. We believe that the predictive role of CRI-Total score depends on the specificity of the questionnaire in evaluating multiple proxies. Indeed, there is evidence that CRI, as a life-experience CR proxy, predicted cognitive performance better than education as a single CR proxy^72^.

Considering the differences in memory trajectories as a function of the accumulation of AD neuropathology, the fact that older adults with higher CR have better recall after Anodal tDCS can have important implications for tDCS interventions to prevent age-related cognitive decline^73^. According to Stern’s hypothetical model of CR^24^, individuals with higher levels of CR can compensate for greater amounts of neuropathology but higher levels of CR are also related to a faster rate of cognitive decline once neuropathology reaches a stage severe enough to affect cognition. So tDCS, if applied in the early stages of the disease, might be an intervention to slow down the rate of memory decline and delay the onset of the symptoms.

Finally, we observed that encoding performance recorded on Day 1 predicted free recall performance on Day 3 and Day 30, irrespective of the tDCS group, suggesting that higher encoding performance scores (more words recalled during the last round of the encoding session) are associated with better free recall performance scores. These results indicate that the number of recalled words was higher if the encoding phase was more efficient and suggests that in healthy aging free recall performance might depend on encoding abilities^74^.

A recent systematic review investigated the inter-individual factors that might influence Anodal tDCS cognitive outcomes (i.e., global cognition and memory) in older adults with and without cognitive impairment^17^. The findings suggest that baseline cognitive function, structural and functional brain imaging, genetic polymorphisms and the use of medications might modulate the effects of tDCS on cognitive outcomes, while cognitive reserve, age, sex, and risk factors for Alzheimer’s disease were not consistently associated to the tDCS effects. However, several factors (i.e. factors related to CR, AD risk co-morbidities, concomitant medications, brain structure and functional connectivity, and genetic polymorphisms) were obtained from a limited number of studies as such, suggesting exercising caution before drawing conclusion. In addition, the sample size of the current work was larger than most of the studies reported in the systematic review^17^ and thus it may not have been underpowered like some of the studies with a smaller sample size. However, further investigations are needed. Regarding limitations, we acknowledge that the sample size of our previous studies was small. Our findings should be reproduced in larger cohorts before clear-cut conclusions can be drawn and to identify all the individual features that might explain response variability, aiming to maximize the therapeutic potential of tDCS to prevent age-related memory decline. Furthermore, longer follow-up visits could be useful in order to deeply investigate the trajectories of memory and the tDCS effects in healthy older adults. Moreover, another limit is represented by the lack of a control stimulation site that should be considered in future studies in order to confirm the specificity of the left PFC for improving episodic memory abilities in healthy aging. Accordingly, other cerebral areas (e.g., temporal, parietal) or different electrode montages, including high-definition tDCS^75^, could also be tested.

Future tDCS studies should examine the complex interactions between different inter-individual factors. In particular, how CR is associated with brain structure and functional connectivity. It is also important to examine the influence of CR on multiple sessions of tDCS that potentially can induce longer-lasting beneficial effects on episodic memory. Finally, we recommend to measure CR with a life experience scales to provide a comprehensive and comparable measurement of the construct^76^.

In conclusion, cognitive reserve measured at baseline with a life experience questionnaire predicts tDCS-induced episodic memory enhancement in older adults and helps to explain response variability in order to design individualized tDCS protocols in the context of precision medicine in the future.

### Supplementary Information

Supplementary Tables.

## Data Availability

All data generated or analyzed during this study are included in this published article and available in a public repository: https://zenodo.org/doi/10.5281/zenodo.7973480.
